# A Good Remedy out of Fashion

**Published:** 1883-10

**Authors:** 


A GOOD REMEDY OUT OF FASHION.

In an address on this subject, delivered at the annual meeting of the Metropolitan Counties Branch of the British Medical Association, by the President, Dr. C. J. Hare, late Physician to University College Hospital, the lecturer made some interesting observations on emetics and bleeding:

"It is not long ago that, in a very urgent case of bronchitis, I advised the administration of an emetic; when the gentleman whom I had been called to meet in consultation said, 'Why, I never gave an emetic to an adult in my life.' In former times it was not unusual, on the contrary, to commence the treatment of many diseases with the administration of a dose to procure vomiting; and although the remedy might then be given sometimes indiscriminately and according to routine, only those who have seen the effects of emetics, properly and judiciously given, can conceive the beneficial effects they sometimes produce. In the early stages of an attack of croup, it was by no means unusual to give an emetic of tartarized antimony or of ipecacuanha; and it is in accordance with the recorded experience of some of the best authorities and most practical men, and quite consonant with my own experience, too, that symptoms which presented the most certain augury of a severe attack were by these means cut short, the hoarse voice resumed its natural character, and the feverish symptoms were in a few hours relieved. I know quite well that a great fear is entertained by some as to the depressing effects of emetics; but the fear is theoretical, and not practical, and those who have had most experience in the administration of them best know how groundless the fear is. In diphtheria, too, I have seen the false membranes which are out of the reach of local remedies, and which the patients cough and cough in vain, and utterly exhaust themselves to get quit of, readily brought up by the action of vomiting, to the immense relief of the sufferer.

"In suffocative bronchitis, the effect of emetics is sometimes magical, and by their administration in such cases not only is immense relief given, but I verily believe—I am certain—that lives are saved. You are called to a patient who has been ill a few days, with increasing dyspnœa; she is sitting up in bed (I draw from nature), for to lie down is impossible; she is restless, and tossing about; the lips, and indeed the whole face, blue; the eyes watery and staring; the pulse quick and small; the cough constant; the expectoration semi-transparent and tenacious; over every square inch of the chest, front and back, from apex to base, you find abundance of rhonchi; moist, sonorous and sibilant ones in the upper part of the lungs, and muco crepitant or mucous *râles* towards the bases. Ammonia and stimulants, right and good in their way perhaps, in such a case are too slow in their action; the patient is, in fact, more or less slowly, more or less rapidly suffocating. An emetic of twenty-two grains of ipecacuanha in an ounce of water is given; in ten or fifteen minutes the patient vomits, and brings up a huge quantity of that tenacious mucus, and the whole aspect of the case is altered; the distressed countenance is relieved; the breathing is at once quieter; and the patient is able for the first time for the past twenty-four hours to lie moderately low in bed, and to get some sweet, refreshing sleep. The patient is, in fact, rescued from the extremest peril, and in this case, and in many similar ones, too, I believe, from otherwise most certain death. Of course, in such cases the emetic is not given for its effect on the stomach, but for its collateral effect in mechanically clearing out the enormous amount of secretion which accumulates in the bronchial tubes, and which the patient is otherwise quite incapable of getting quit of; and thus the half choking, almost asphyxiated condition is changed for one of comparative comfort, and time is gained for the action of other appropriate remedies. No doubt the secretion may, and often will, accumulate again ; and I have not hesitated again in bad cases to repeat the same good remedy ; but it is a fact, and a very positive one, too, that, quite contrary to what those who have had no experience in the plan suppose, the system rallies instead of being more depressed under the action of the remedy."—*British Medical Journal.*

